# History of *Taenia saginata* Tapeworms in Northern Russia

**DOI:** 10.3201/eid2312.162101

**Published:** 2017-12

**Authors:** Sergey V. Konyaev, Minoru Nakao, Akira Ito, Antti Lavikainen

**Affiliations:** Siberian Branch of the Russian Academy of Sciences, Novosibirsk, Russia (S.V. Konyaev);; Asahikawa Medical University, Asahikawa, Japan (M. Nakao, A. Ito);; University of Helsinki, Helsinki, Finland (A. Lavikainen)

**Keywords:** Taenia saginata, tapeworms, Taenia spp., taeniasis, cestodes, parasites, zoonoses, reindeer, brain, indigenous people, northern Russia, Russia, Siberia, Arctic

## Abstract

*Taenia saginata* is the most common species of tapeworm infecting humans. Infection is acquired by eating cysticercus larvae in undercooked beef. A closely related species, *T. asiatica*, is found in eastern and southeastern Asia. The larvae of *T. asiatica* develop in viscera of pigs. In northern Russia, there is a third member of this morphologically indistinguishable group. Cysticerci of so-called northern *T. saginata* are found in cerebral meninges of reindeer, and the unique life cycle is dependent on a native custom of eating raw reindeer brain. We report the winding history of this mysterious tapeworm from the first reports to the present time. In addition, we confirm the position of this parasite as a strain of *T. saginata* by analyzing a mitochondrial DNA sequence of an archival specimen. The origin of this strain might date back to reindeer domestication and contacts between cattle-herding and reindeer-herding peoples in Asia.

Taeniasis is among the oldest known human helminthiases; written descriptions of the disease and history of the name taenia reach into antiquity. *Taenia* spp. infections are common in many countries; there are tens of millions of human carriers worldwide (*1*). The best-known etiologic agents, *Taenia saginata* Goeze 1782 and *T. solium* Linnaeus 1758, were described as 2 different tapeworm species >2 centuries ago, and both have many synonyms in the literature (*2*). Conversely, a third species of *Taenia* infecting humans, *T. asiatica* Eom et Rim 1993, is one of the most recently described taeniid species. This species was known to be a special form of *T. saginata* (*3*).

Similar to other taeniid tapeworms, human-infecting *Taenia* spp. require 2 mammalian hosts in an obligate predator–prey life cycle. Adult tapeworm stages develop in the human intestine. Gravid proglottids, which are full of eggs, are excreted in feces into the environment. Alternatively, for *T. asiatica* and *T. saginata*, proglottids can actively crawl out of the anus and cause irritation and discomfort (*1*–*3*). The infection is otherwise usually asymptomatic. Eggs scattered in the environment are then ingested by intermediate hosts, cattle and other bovids (for *T. saginata*) or pigs (for *T. asiatica* and *T. solium*). Cysticercal larvae typically develop in muscles (*T. saginata* and *T. solium*) or visceral organs (*T. asiatica*) of the intermediate host. Humans can become infected by eating raw or undercooked meat or organs infected with cysticerci.

Unlike the other 2 species, *T. solium* commonly forms cysticerci in tissues of various atypical intermediate hosts, including rabbits, camels, dogs, cats, and humans (*2*,*4*). Another major feature distinguishing *T. solium* from the other 2 species is a double crown of rostellar hooks, which can be easily observed by microscopy in adult and larval stages; these hooks are absent in *T. asiatica* and *T. saginata*.

*T. saginata* is the most common and widely spread *Taenia* species infecting humans. This tapeworm is found in all continents and is endemic to eastern Europe, Southeast Asia, Africa, and Latin America (*1*,*5*). However, in addition to the classic strain of this parasite found in southern regions, which is associated with cattle raising, there is a lesser-known form of *T. saginata* in northern regions. Its present distribution is limited, perhaps including only some parts of northern Russia. This northern form or strain of *T. saginata* uses reindeer (*Rangifer tarandus*) instead of bovids as the intermediate host. The aim of this article is to provide information on the history and unique life cycle of this enigmatic human parasite in northen Russia and to resolve its taxonomic position on the basis of unpublished DNA data.

## Early Records of *T. saginata* Infections in Reindeer and Reindeer-Herding Human Populations

**Figure 1:**
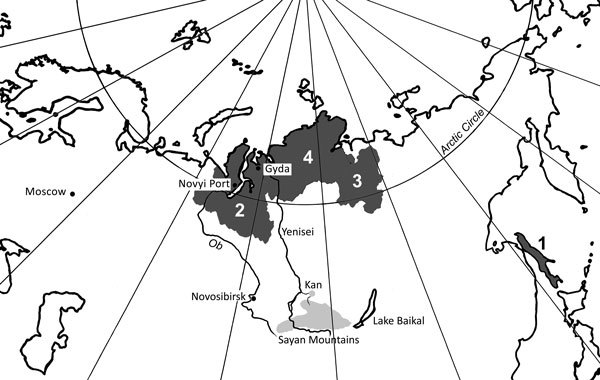
Regions where infections with the northern strain of *Taenia saginata* tapeworms in Siberia and the Far East region of Russia were detected (dark gray shading). 1, Sakhalin Island in 1872 and 1955; 2, Yamalo-Nenets Autonomous Okrug, since the 19th century to the present time; 3, Olenyoksky District, Yakutia, 1958; 4, Taymyr Autonomous Okrug, 1977. The probable cradle of reindeer herding in Asia and site of the host switch from cattle to reindeer (the Sayan Mountains) is indicated by light gray shading. The light gray dot indicates the Kan River archaeologic site where taeniid eggs were found from human remains buried 3,000–4,000 years ago.

The northern strain of *T. saginata* has been found in northern Siberia in Russia and the Far East Region of Russia (Figure 1). Krotov (*6*,*7*) reported that the northern strain of *T. saginata* was observed in 1872 by Dobrotvorsky, who reported taeniasis in the native population of Sakhalin Island. At that time, cattle had not yet been brought to the island. This finding was supported by observations of Krotov on taeniasis in reindeer herders on Sakhalin Island in 1955 (*7*). Researchers considered reindeer to be the most likely intermediate host, although larval stages were not found (*7*). Unfortunately, we could not analyze details of this study because we did not have access to the original data of Krotov, which were published in his academic dissertation in 1955. After the observations of Krotov, taeniasis on Sakhalin Island did not attract further attention.

The suggestion that cases of taeniasis on Sakhalin Island represent the first report of the northern strain of *T. saginata* cannot be verified. The current population of Sakhalin Island has essentially abandoned reindeer herding. Reindeer are still used, but their number, as well as the size of the indigenous human population, is small. Large-scale reindeer husbandry on Sakhalin Island began in the Val collective farm (*8*), where Krotov (*7*) later detected possible human taeniasis. The reindeer population was initially 414 animals in 1930 and increased to 8,415 by 1967 (*8*). However, by 1998, after the collapse of the Soviet Union, the number of reindeer had decreased to 2,900. The present population is probably 150–200 animals.

Only the indigenous Ulta, which have a population of <350 persons (*8*), maintain the reindeer herding tradition on Sakhalin Island. Given the sharp decrease in the number of intermediate and final hosts, the northern strain of *T. saginata* has probably not survived on Sakhalin Island. In addition, a return to small-scale reindeer herding indicates that domestic reindeer are used primarily as mount or pack animals, and wild reindeer are consumed as food. According to a state report of human parasitoses on Sakhalin Island, no cases of taeniasis have been found there in recent years (*9*).

In 1956, taeniasis in an indigenous person in the Krasnoselkup region in the Yamalo-Nenets Autonomous Okrug (YaNAO) caused concern about spread of *T. saginata* in northern Siberia (*10*). The index patient was a Selkup reindeer herder. Anthelminthic treatment for 32 tapeworm carriers identified 11 cases of taeniasis among reindeer herders. Diphyllobothriasis and taeniasis were distinguished, but morphologic data for tapeworms were not provided in detail. It was concluded that the source of infection was probably reindeer meat, which was (and still is) the staple food in that region (*10*). Subsequently, taeniasis in humans was confirmed across the YaNAO (including the basins of the Ob, Nadym, Pur, and Taz Rivers and the Yamal and Gydan Peninsulas), in Khanty-Mansiysk (the capital of the Khanty-Mansi Autonomous Okrug); and in the Taymyr Autonomous Okrug (*11*–*17*). The highest prevalence (14%) in the local population was reported in the Yamal and Gydan Peninsulas (*14*). A recent archaeologic finding of taeniid eggs in a burial site from the 19th century (*18*) showed that taeniasis occurred in the Nenets population in the Taz tundra, which was contemporary with the first observation on Sakhalin Island (*7*).

In 1958, possible cysticerci of *T. saginata* were found in reindeer carcasses and organs in the Oleneksky District, Yakutia, but a specific diagnosis was not confirmed by microscopy (*19*). A survey identified tapeworm infection in 59 of 200 local schoolchildren (*19*). The local population consumed reindeer meat and fish. Nevertheless, infection with *Diphyllobothrium latum* tapeworms was not considered a possible differential diagnosis. At that time, beef was not consumed in the Oleneksky District (*19*). However, cattle were raised in other parts of Yakutia south of the Oleneksky District. Overall, the presence of reindeer-dependent *T. saginata* in Yakutia cannot be reliably confirmed. Recent records (*20*) showed that all recent cases of taeniasis reported in Yakutia were associated with beef consumption. Despite extensive meat inspection, cysticerci of *T. saginata* have not been currently reported in reindeer in Yakutia.

In a monograph on taeniid taxonomy and biology, Abuladze (*2*) summarized data on *Taenia* spp. circulating in northern Russia. He speculated that a special species of the genus *Taeniarhynchus* (in his classification, *Taenia* spp. without rostellar armature were considered a distinct genus) might be involved. However, this suggestion did not lead to a description of the full morphology or individual segments of the putative new species (*2*).

## Experimental Infections

**Figure 2:**
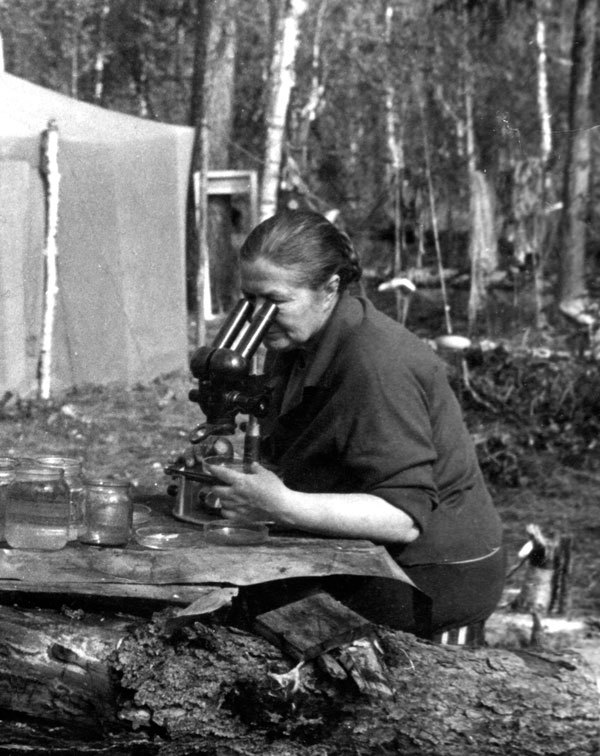
Vera I. Shakhmatova, one of the pioneers of infection experiments with the northern strain of *Taenia saginata* tapeworms, examining parasitologic specimens during a field expedition in northern Siberia, Russia, 1975. Photograph courtesy of the Institute of Systematics and Ecology of Animals, Siberian Branch of the Russian Academy of Sciences.

The first study to resolve the life history and host specificity of the northern strain of *T. saginata* was conducted in 1975 by Mozgovoy et al. at the Biological Institute (currently the Institute of Systematics and Ecology of Animals) of the Academy of Sciences, Novosibirsk (*16*) (Figure 2). A limited number of reindeer and cattle calves were infected with the southern and northern strains of *T. saginata* (Technical Appendix Table 1). Necropsies were performed on the animals after various postinfection periods. The southern strain was obtained from a patient in Barnaul (Altay Krai), and the northern strain was obtained from a patient in Gyda (a remote village in YaNAO).

Many fibrotic nodules, but no *T. saginata* cysticerci, were observed in muscles of 5 reindeer calves. One reindeer died because of a heavy infection with the northern strain. Numerous cysticerci were found in muscles, heart, diaphragm, and brain of this calf. Infectivity of larvae for humans was not tested, but ≈60% of muscle cysticerci were dead (necrotic). In a reindeer infected with the southern form, cysticerci were found only in muscles. However, the experiment was subject to environmental contamination because animals were not isolated from the herd. Armed (containing rostellar hooks) cysticerci of *Taenia* spp. transmitted by dogs (e.g., *Taenia parenchymatosa* tapeworms) were found in 6 reindeer, which indicated contamination of the pasture. This finding indicated that all experimental animals could also have had access to eggs of the northern strain of *T. saginata* excreted by herdsmen.

**Figure 3:**
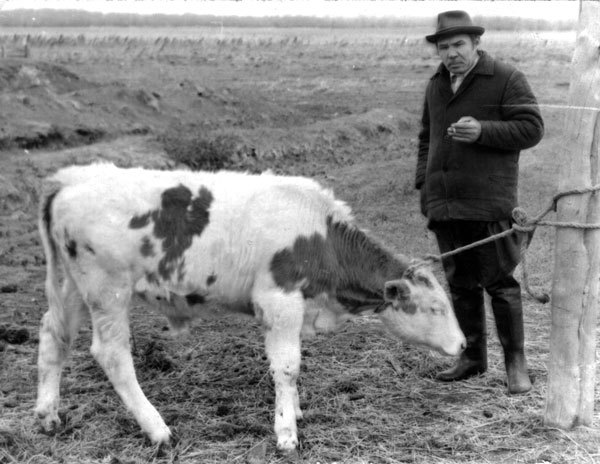
Researcher Anatoly M. Serdyukov with a calf experimentally infected with *Taenia saginata* tapeworms, Sovkhoz Rossia, Altai Krai, western Siberia, Russia, 1975. Photograph courtesy of the Institute of Systematics and Ecology of Animals, Siberian Branch of the Russian Academy of Sciences.

**Figure 4:**
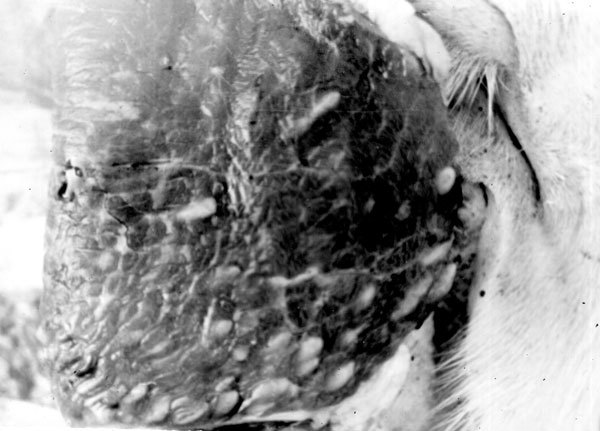
Muscle cysticerci of the northern strain of *Taenia saginata* tapeworms in an experimentally infected calf, western Siberia, Russia. Photograph courtesy of the Institute of Systematics and Ecology of Animals, Siberian Branch of the Russian Academy of Sciences.

Infected cattle calves were kept in isolation in Altay Krai (Figure 3). Cysticerci of northern and southern strains developed in muscles (Figure 4). However, 55%−90% of cysticerci of the northern strain were found dead. Viability of larvae was confirmed by self-infection. One of the authors (not identified) of the study of Mosgovoy et al. (*16*) ate 4 cysticerci of the northern strain and shed *T. saginata* proglottids 80 days postinfection (Technical Appendix Table 2).

These experiments yielded 2 conclusions. First, larvae of the northern strain can develop not only in muscles but also in the brain of reindeer. Second, the northern strain is capable of developing in cattle, although viability seems to be drastically reduced. However, this pioneering work went unnoticed by the Soviet scientific community, and researchers in Moscow later conducted similar experiments.

A research group from the K.I. Skrjabin Institute of Helminthology in Moscow conducted a series of experiments during the 1980s (*17*,*21*,*22*) that essentially duplicated the previous study conducted by Mozgovoy et al. (*16*). Eggs of the northern strain were obtained from 2 Nenets children from a small settlement (Novyi Port) on the Yamal Peninsula (*17*,*21*). The only meat the 2 children consumed was reindeer meat, and the children never left the territory. Comparative specimens for the southern strain were obtained from Moscow and Uzbekistan. Results confirmed the previous findings of Mozgovoy et al. (*16*). On the basis of a slightly higher number of experimental animals (Technical Appendix Table 1), the affinity of the northern strain for reindeer brain was demonstrated. Low viability in cattle was also shown. In addition, 3 naturally infected reindeer were found among 413 carcasses examined in Novyi Port (*17*). All infected reindeer were >2 years of age (this group included 201 animals), and 1 or 2 cysticerci of *T. saginata* per animal were found (all in the brain). This finding was probably the only confirmed observation of natural infections in reindeer.

During an initial experiment, Kirichek et al. reported that “two reindeer died 14 and 28 days after infection without visible clinical signs of cysticercosis” (*17*). However, there was a dramatic story behind this laconic sentence. Reindeer used for experiments were born and reared isolated from natural herds in a vivarium in Salekhard (*17*). According to an anonymous eyewitness, the infected reindeer (2 adults and 1 calf) were transported to Moscow for observation. Just after arrival, both adult reindeer escaped. One soon died in a car accident, but the other one ran away and spent 2 weeks in parks in Moscow, until it was found and killed. These reindeer were unlikely to have been exposed to additional *T. saginata* eggs during their escape. More than 1,000 degenerating cysticerci were found in their bodies. However, heads of these animals were not examined, possibly because of unscheduled dissections (*17*).

In 1986, successful self-infection was performed by Kirichek et al. with cysticerci from the brain of an experimentally infected reindeer (*21*) (Technical Appendix Table 2). Cysticerci, collected 115 days postinfection were infective. The clinical course, including the prepatent period (97 days), signs (periodic diarrhea, light meteorism [rapid accumulation of gas in the intestine], anal irritation caused by actively moving proglottids, pyrosis, vomiting, and lack of appetite), and excretion of proglottids (*21*), corresponded to that of taeniasis caused by *T. asiatica* or the beef-derived southern strain of *T. saginata* (*1*–*3*).

## Cerebral Cysticerci and Morphologic Characterization of Adult Tapeworms

Histopathologic analysis of northern strain cysticerci was performed by parasitologists from the Soviet Union and Czechoslovakia (*22*) for specimens from the experimental studies of the K.I. Skrjabin Institute (*17*,*21*). Viable cysticerci in reindeer were located in the subarachnoid space but were not found in brain tissue or spinal cord (*22*). Larval structure and tissue reactions corresponded to those of the southern strain in cattle muscle (*22*). The infection typically caused a nonpurulent meningoencephalitis with neurologic symptoms (e.g., reeling and walking in circles) (*17*). Cysticerci in reindeer muscles and heart died at an early developmental stage, possibly because of nutritional deficiency and immunologic response, and were fully resorbed within 3 months (*17*,*22*). It was assumed that immunologic responses appear later in the brain than in other tissues or are less effective (*22*).

**Figure 5:**
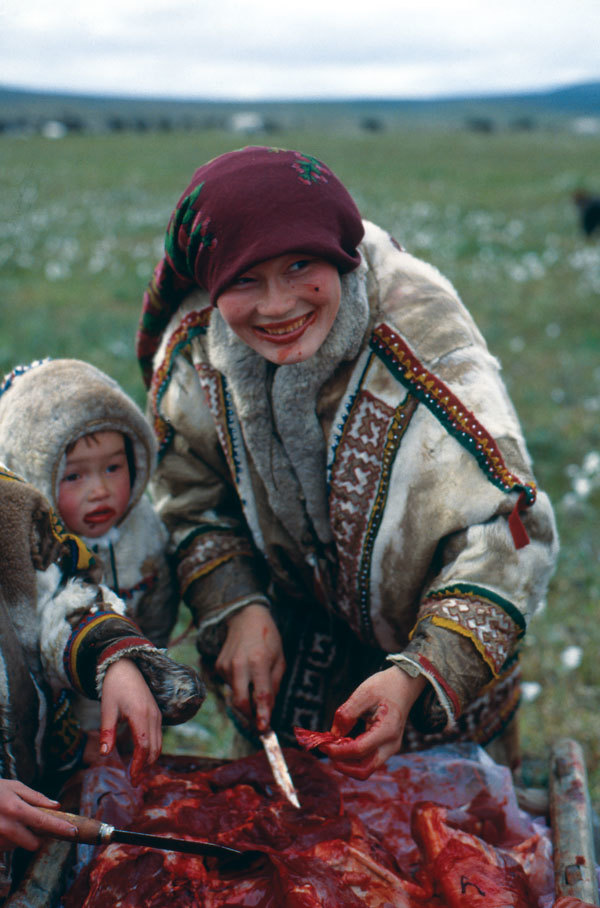
A Khanty mother and her child eating raw reindeer meat in the Yamal Peninsula (Yamalo-Nenets Autonomous Okrug), northern Siberia, Russia, 1991. Photograph courtesy of Marianna Flinckenberg-Gluschkoff.

Cerebral localization of cysticerci explained the unsuccessful attempts of previous researchers to find larvae of *T. saginata* in reindeer carcasses. The nonindigenous population in northern Siberia eagerly consumes venison, often undercooked. However, raw brain is consumed only by the native population. Given the extremely limited natural resources in northern Siberia, raw brain and other tissues have traditionally been a common source of nutrition for many indigenous peoples in this region (Figure 5).

**Figure 6:**
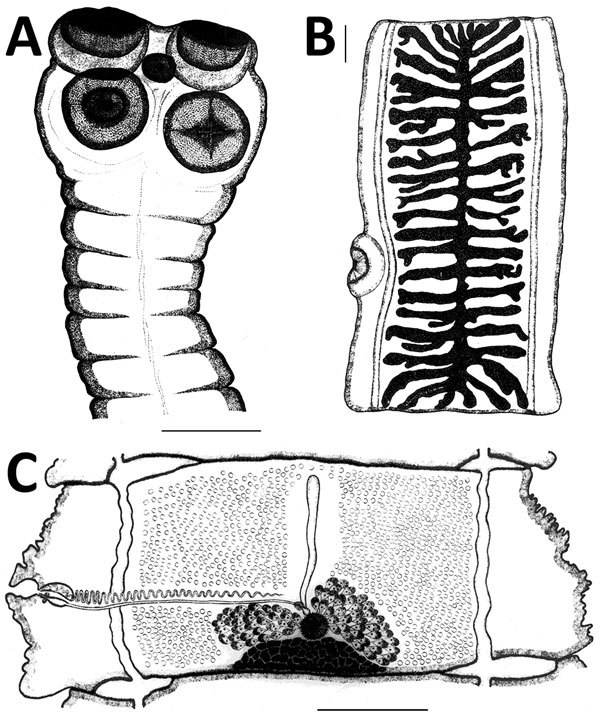
Original drawings of the northern strain of *Taenia saginata* tapeworms from Gyda, Yamalo-Nenets Autonomous Okrug, northern Siberia, Russia, by Serdyukov (*23*). A) Scolex showing the lack of a rostellar hook crown in the middle of the scolex, which is a synapomorphy in *T. saginata* and *T. asiatica* tapeworms. B) Gravid proglottid showing the number of uterine branches, which is a commonly used character in species identification. C) Mature proglottid. Scale bars indicate 1 mm. Image courtesy of the Institute of Systematics and Ecology of Animals, Siberian Branch of the Russian Academy of Sciences.

Morphologic studies of northern *T. saginata* adult stages are limited. The literature does not provide data describing detected cestodes but refers only to their specific identification. It seems logical that expelled fragments were identified after diagnostic deworming, but criteria were not provided. We must assume that diagnoses were primarily based on 2 widely used attributes: lack of hooks in the scolex and number of uterine branches in mature proglottids (Figure 6, panels A, B).

A complete morphologic description of the northern strain was reported (*23*) (original illustrations in Figure 6). Serdyukov (*23*) also reported a detailed comparison between northern and southern strains by using specimens from patients in Gyda (8 specimens) and Novosibirsk (4 specimens). This study was conducted simultaneously with the experimental work of his colleagues (*16*). Only a few minor differences were detected (e.g., the cirrus sac was longer and the average number of uterine branches was slightly higher in the southern strain). Given the poorly understood natural phenotypic variation, the role of these differences remains unclear. If one considers the nearly complete congruence of morphologic characteristics, specimens of the northern strain were clearly related to *T. saginata* (and *T. asiatica*) and could not represent any other known species or group of *Taenia* tapeworms.

## Identification by DNA Barcoding

In the Soviet Union and later in Russia, the northern strain of *T. saginata* was considered a form or isolate confined to an atypical intermediate host, the reindeer. This definition has not been subjected to further questioning, beyond the unsupported contention that it might represent a distinct species (*2*). Since the description of *T. asiatica*, a species closely related to *T. saginata*, a taxonomic reconsideration of the northern strain has become relevant. Nonbovid intermediate hosts and distinctly different predilection sites for cysticerci differentiate *T. asiatica* and northern *T. saginata* from classic *T. saginata*. Because *T. asiatica* was not known by parasitologists of the Soviet era, host switching from pigs to reindeer was not considered, and cysticercosis in pigs was not investigated in disease-endemic areas. Pigs are commonly reared for food production in villages in northern Russia because they are much easier to keep than cattle during the cold season.

A major question is the relationship between the northern strain and other unarmed (no rostellar hooks) *Taenia* spp. Could it represent an independent lineage, or could it be related to *T. asiatica* instead of classic *T. saginata*? On the basis of available descriptions (*23*–*25*), all 3 forms are virtually morphologically indistinguishable. Thus, molecular genetic characterization was required to confirm the identity of the northern strain.

To resolve this question, we analyzed a formalin-preserved archival specimen of the northern strain from the collection of the Institute of Systematics and Ecology of Animals in Novosibirsk. A fully developed tapeworm was expelled from a 7-year-old Nenets child in Gyda in 1974. The patient had never consumed beef or pork. Morphology of the specimen was reported (*23*). In addition, we attempted to extract DNA by using cysticerci (all specimens were preserved in formalin) from reindeer obtained during the experiments of Mosgovoy et al. (*16*), but PCR amplifications were unsuccessful.

The specimen from Gyda was characterized on the basis of a region of the mitochondrial cytochrome c oxidase subunit 1 (*cox1*) gene of *T. saginata* (mitochondrial genome DDBJ/EMBL/GenBank accession no. NC_009938), which has been used for >20 years in barcoding of taeniids. We isolated genomic DNA from a tapeworm proglottid by using the DNeasy Tissue Kit (QIAGEN, Hilden, Germany). Because the DNA was fragmented and damaged by prolonged preservation in formalin, we generated short overlapping amplicons (≈200 bp each) by using specific primer pairs (Technical Appendix Table 3). We performed PCR with 40 thermal cycles (94°C for 30 s, 50°C for 30 s, and 72°C for 30 s) and cloned amplicons by using the pGEM-T Vector System (Promega, Madison, WI, USA). Procedures for PCR, cloning, and sequencing have been reported (*26*). Overlapping *cox1* sequences obtained were assembled into a single contiguous sequence (426 nt, DDBJ/EMBL/GenBank accession no. LC063349). The resultant sequence was phylogenetically compared with those of *T. saginata*, *T. asiatica*, and *T. crocutae* (an outgroup). We used MEGA6 software (*27*) for alignment, distance calculation, substitution model search, and maximum-likelihood estimation.

**Figure 7:**
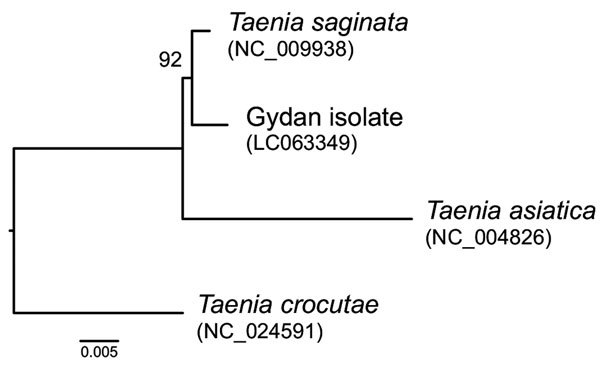
Maximum-likelihood tree of *Taenia saginata* tapeworm strains and *T. asiatica* tapeworms inferred from short fragments (426 nt) of the cytochrome c oxidase subunit 1 gene. The northern strain of *T. saginata* tapeworm is represented by the Gydan isolate from northern Siberia, Russia. *T. crocutae* tapeworm was used as an outgroup. Number along branch indicates a bootstrap percentage. GenBank accession numbers of original sequences are shown in parentheses. Scale bar indicates nucleotide substitutions per site.

The sequence of *cox1* suggested that the specimen of the northern strain represents *T. saginata* (Figure 7). The *cox1* fragment differed only by 3 nt from the corresponding reference sequence of *T. saginata.* Nevertheless, no sequences identical to the Gydan specimen were found in DNA databases. The difference was within the variation that can be detected for sequences of *T. saginata* in these databases (DDBJ/EMBL/GenBank). Unfortunately, a short sequence from 1 specimen does not enable estimation of phylogeographic relationships of the northern strain.

*Taenia asiatica* and *T. saginata* are considered distinct species mainly because of differences in life history, localization in the intermediate host, and phylogenetic data indicating a sister species relationship (*24*,*28*,*29*). However, genetic studies have shown recent hybridization of these parasites, which clearly indicates that the reproductive barrier between them is not complete and is consistent with a relatively shallow time frame for divergence (*30*). This finding makes the validity of *T. asiatica* as a separate species questionable. Genetic distance between *T. asiatica* and *T. saginata* is much higher than between the northern and southern strains of *T. saginata* (Kimura 2-parameter distance 0.039 vs. 0.007, respectively). The northern strain of *T. saginata* is partially adapted (by unknown mechanisms) to the reindeer intermediate host (*17*,*22*). Evidence suggests a recent origin of the human–reindeer cycle in this geographically restricted area. Therefore, we conclude that strain is the most suitable definition for this parasite, as well as the basically equal form, which was used by parasitologists in the Soviet Union. The term isolate, which has also been used in Russia for *T. saginata* from northern regions, today refers most often to a particular taeniid specimen or materials derived from it.

## Origin and Current Situation

The earliest archaeologic evidence of reindeer domestication comes from the Sayan Mountains on the border of Siberia and Mongolia and dates from >2,000 years ago (*31*,*32*) (Figure 1). Reindeer domestication was not an isolated process, but in relation to other domesticated animals, including cattle (*31*,*32*), domestications provided an interface for host switching of *T. saginata*. A recent archaeoparasitologic study in the region of the Kan River, which runs northward from the Sayans Mountains, reported that human-infecting *Taenia* tapeworms might have established a wildlife-dependent cycle in Siberia before reindeer domestication (*33*). Three taeniid eggs were found in remains of a human buried 3,000–4,000 years ago, but animal bone findings at archaeological sites showed that diet during that period was based mainly on cervids other than reindeer (*33*). *T. saginata* is not known to have a life cycle involving wild cervids.

Reindeer herding (and probably related parasites) spread widely across Siberia. Dominating cultures have later repressed old food habits. For example, in Yakutia, the Yakuts, who came relatively recently from the south, do not consume raw brain, but the Evenki, who are descendants of the first reindeer herders who migrated north and northeast from the region of Lake Baikal (*32*), still continue to locally maintain old dietary practices.

Genetic evidence indicates that reindeer were domesticated independently in Fennoscandia (northern Europe) (*34*). The indigenous reindeer-herding Sámi people do not eat raw reindeer tissues and have not done so in recent centuries (*35*,*36*). In Finland, for example, the traditional Sámi diet included reindeer brain in cooked brain cakes; only blood was occasionally consumed raw to provide health benefits (*36*). These findings indicate that there was no ecologic niche for a brain-associated parasite in the western part of the reindeer husbandry area of Eurasia, or it became extinct a long time ago.

Today, YaNAO appears to be the only region to which the northern strain of *T. saginata* is endemic. Epidemiologic studies have shown that 10–25 cases are reported annually in YaNAO; virtually all cases are linked to raw reindeer brain consumption (*37*,*38*). Most of these cases were identified in the native population, which is deeply committed to traditions. Tapeworm carriers are typically nomadic reindeer herders.

Approximately 50,000–60,000 reindeer are slaughtered annually in YaNAO (*37*–*39*). Reindeer heads are not used for commercial food production or otherwise by industry. Thus, reindeer brains are not routinely inspected for *T. saginata* cysticerci. According to the regional government of YaNAO, the reindeer population of the district is 730,000 animals, which is the largest herd in the world (*39*,*40*). More than 16,000 persons are involved in reindeer herding and migrate on the tundra. These persons have traditional nomadic lifestyles, food habits, and minimal access to healthcare. These facts, together with the long life span of the parasite, ensure that the northern strain of *T. saginata* will survive in this region for many years. Nevertheless, the modern world (globalization, oil drilling, and climate change) casts a dark shadow over the parasite life cycle, which is dependent on fragile traditional cultures of native populations in these regions.

Technical Appendix Additional information on the history of *Taenia saginata* tapeworms in northern Russia.
