# Supplementary material for: History of Taenia saginata Tapeworms in Northern Russia
Source: Emerg Infect Dis. 2017 Dec;23(12):2030–7. doi: 10.3201/eid2312.162101 (PMC5708244; doi:10.3201/eid2312.162101)
Supplement: Technical Appendix — Additional information on the history of Taenia saginata tapeworms in northern Russia. [file 16-2101-Techapp-s1.pdf]

Article DOI: https://doi.org/10.3201/eid2312.162101

History of *Taenia saginata* Tapeworms in Northern Russia

Technical Appendix.

Technical Appendix Table 1. Experimental infections of animals with strains of *Taenia saginata* tapeworm, Russia*

Ref.	Animal	No. infected	Strain	Infective dose of eggs	Signs (no. animals)†	No. deaths	Time of examination, dpi‡	Cysticerci in muscle or visceral organs§		Cysticerci in brain§	
								No.	% Alive	No.	% Alive
1	Cattle	2	S	300,000–350,000	General (2)	1	22–138	+	++	0	NA
1	Reindeer	5	S	300,000–350,000	Transient (5)	0	60–105	30 (1 animal; fibrotic nodules in others)	?	0	NA
1	Cattle	3	N	300,000–350,000	General (3)	2	25–135	+	10–45	0	NA
1	Reindeer	1	N	300,000–350,000	Transient (1)	0	105	(fibrotic nodules)	0	0	NA
1	Reindeer	1	N	400,000	General; cachexia (1)	1	30	Max. 9–16/g	40	+	++
2	Cattle	1	S	10,000	?	0	50	87	75	0	NA
2	Reindeer	4	S	5,000	?	0	25–90	0–7	0	0	NA
2	Cattle	3	N	10,000	?	0	47–60	473–597	2–8	0	NA
2	Reindeer	7	N	5,000–10,000	Neurologic (?)	0	14–90	0–1,048	0–1	8–47	98–100
3,4	Reindeer	1	N	?	?	0	115	0	NA	+	?

*Experimental animals were individually infected. *dpi, days postinfection; N, northern strain; NA, not applicable; Ref., reference; S, southern strain; ?, no data provided.
†General: malaise, depression, fever, or other signs; transient: malaise, depression, and tachypnea.
‡Animals were examined by using gross necropsy and microscopy.
§+, cysticerci found but not counted; ++, numerous living cysticerci but their proportion was not estimated.

Technical Appendix Table 2. Experimental infections of humans with northern strain of *Taenia saginata* tapeworm from experimentally infected animals, Russia*

Ref.	No. infected	Dose of cysticerci	Origin of cysticerci, dpi	Prepatency, d†,	No. expelled worms
1	1	4	Cattle, 135	80	?
3	1	2	Reindeer, 50	NA	0
3	1	3	Reindeer, 115	97	3

*dpi, days postinfection; NA, not applicable; Ref., reference; ?, no data provided.
†Infections were diagnosed by observing proglottids of *T. saginata* in feces.

Technical Appendix Table 3. Primer sets used in amplification of overlapping fragments covering 426 nt sites of the mitochondrial cytochrome c oxidase subunit 1 gene of an archival specimen of the northern strain of a *Taenia saginata* tapeworm, northern Russia

Set	Forward primer, 5′→3′	Reverse primer, 5′→3′
A	GGT GAT CCT GTT TTA TTT CAA CAT ATG	CCA ACC GTA AAC ATA TSA TGA CCC CAC
B	TTT GTT RTT TGC TAT GTT TTC AAT AGT	CTT ATT TAC ACG AGA ATT TAA AAG CAT
C	ATA GGA GTA CCA ACA GGA ATA AAG GTT	ACC CTA AYG ACA TAA CAT AAT GAA AAT G

References

1. Mozgovoy AA, Koval’chuk ES, Gosteev AK, Shakhmatova VI. Study of some population characteristics of *Taenia saginata* in western Siberia [in Russian]. Proceedings of the Biological Institute of the Siberian Branch of the Academy of Sciences. 1979;38:190–205.

2. Kirichek VS, Belousov MN, Nikitin AS. New data on the epidemiology of taeniarhynchiasis in regions of the Far North in the USSR (based on observational data in the Yamal-Nenets Autonomous Okrug) [in Russian]. Med Parazitol (Mosk). 1984;Nov–Dec:27–33. PubMed

3. Kirichek VS, Nikitin AS, Frolova AA, Iarotskiĭ LS. Various features of the biology of a northern isolate of *Taeniarhynchus saginatus* Coeze, 1782 [in Russian]. Med Parazitol (Mosk). 1986;Nov–Dec:37–9. PubMed

4. Blažek K, Kirichek VS, Schramlová J. Pathology of experimental *Cysticercus bovis* infection in the reindeer (*Rangifer tarandus* Linné, 1758). Folia Parasitol (Praha). 1986;33:39–44. PubMed
